# Comparison of the Volatile Components of *Apocynum venetum* Honey from Different Production Areas in Xinjiang

**DOI:** 10.3390/foods14223860

**Published:** 2025-11-11

**Authors:** Na Zhang, Jingjing Lv, Ruili Zhang, Beibei Sun, Ning Du, Yawen Li

**Affiliations:** 1 College of Food Science and Engineering, Tarim University, Alar 843300, China; zhangna@taru.edu.cn (N.Z.); 13333973910@163.com (J.L.); 2 Production & Construction Group Key Laboratory of Special Agricultural Products Further Processing in Southern Xinjiang, Alar 843300, China; 3 Instrumental Analysis Center, Tarim University, Alar 843300, China; zrl_p@sina.com (R.Z.); beibeisun0410@163.com (B.S.); duing1@yeah.net (N.D.)

**Keywords:** *Apocynum venetum* honey, different origin, HS–SPME–GC–MS, volatile components

## Abstract

*Apocynum venetum* honey, a characteristic Chinese herbal honey, is a key agricultural product in Xinjiang. To better understand its unique flavor and geographical authenticity, this study analyzed the volatile components of honey samples from three production regions via headspace solid-phase microextraction combined with gas chromatography–mass spectrometry. Overall, 160 volatile compounds were identified, with 34 exhibiting aroma activity values of >1. Notably, chemometric analysis revealed 24 key differential compounds, including phenylethyl alcohol, benzyl alcohol, 2-furanmethanol, 5-ethenyltetrahydro-α,α,5-trimethyl-, cis-, cedrol, 2,4-di-tert-butylphenol, decanal, and nonanal, which significantly contributed to both geographical discrimination and unique flavor profiles. Cluster heatmap analysis demonstrated that these markers could be used to effectively differentiate the samples by origin. The research results provide a theoretical basis for the further development and utilization of this honey as well as support for expanding honey resources for use in traditional Chinese medicine.

## 1. Introduction

*Apocynum venetum* L. belongs to the Apocynaceae family and is widely distributed across northern China, with Xinjiang being its primary growing region. This plant species is known for its medicinal properties, including antioxidant, anti-inflammatory, and cardioprotective effects [1,2,3,4]. *A. venetum* honey, which is produced by bees that mainly feed on *A. venetum* nectar, is valued in traditional Chinese medicine for its lung-moistening and heart-strengthening properties [5,6,7,8] and is a key economic product for Xinjiang’s beekeeping industry [9]. However, the volatile composition of *A. venetum* honey sourced from this region remains uncharacterized.

The special aroma of *A. venetum* honey is attributed to its volatile profile, with >600 volatile components being identified to date [10,11]. The production region of honey influences its volatile profile, which is responsible for the unique aroma of honeys from different geographical areas. Furthermore, the volatile profile of honey is not only dependent on its nectar source but also on its processing and storage as well as other factors [12,13]. Headspace solid-phase microextraction combined with gas chromatography–mass spectrometry (HS–SPME–GC–MS) is a cost-efficient and highly effective and sensitive technique for honey extraction. In addition, it has been widely employed to identify honey species and their origin [14,15,16]. Yang Zhen et al. [17] identified 147 volatile organic compounds in hazelnut using HS–SPME–GC–MS, revealing the key metabolic changes that lead to the development of the hazelnut flavor. Karabagia et al. [18] employed this method to study thyme from the five main production areas in Greece and applied chemometric methods to qualitatively and semi-quantitatively analyze the volatile components (methyl formate, ethyl decanoate, formic acid, and acetic acid) of the different thyme samples. They found that nine volatile components could be used as markers to identify the geographical origin of thyme. The volatile components of gallnut honey collected from six regions in Chongqing were analyzed and identified using HS–SPME–GC–MS combined with principal component analysis (PCA) [19]. The results showed that nonanal and cedrol were responsible for the formation of unique waxes and fragrances in gallnut honey from Yunyang and Shizhu, respectively, while damascenone imparted apple, rose, and sweet flavors to gallnut honey from the other four regions. Thus, previous studies have shown that HS–SPME–GC–MS is an established method for characterizing the volatile components of honey. Additionally, the present study uses various chemometric analyses combined with heat maps to analyze the volatile components of *A. venetum* honey, making the data more convincing. This study aimed to identify and compare the volatile components of *A. venetum* honey from three different regions in Xinjiang using HS–SPME–GC–MS and evaluate their potential as geographic markers.

## 2. Materials and Methods

### 2.1. Materials and Reagents

To ensure the authenticity of the *A. venetum* honey samples used in this study, they were directly collected from beekeepers at the full-bloom stage. The samples were collected in September 2024 from three districts and counties in Xinjiang, with five samples obtained from each location: AKS1-5 (Aksu), KS1-5 (Kashgar), and BZ1-5 (Bayinguoleng Mongolian Autonomous Prefecture). The sampling points were selected from the main industrial regions of the study area to ensure that they accurately represent the characteristics of the area. Simultaneously, we selected sampling in regions upwind of (or away from) pollution. After collection, the samples were filtered, canned, and stored at 4 °C. Sodium chloride (analytically pure) was purchased from Zhiyuan Chemical Reagent Co., Ltd., Tianjin, China, and cyclohexanone (high-performance liquid chromatography [HPLC]-grade) was purchased from McLean Biochemical Technology Co., Ltd., Shanghai, China.

### 2.2. Equipment and Instruments

The TSQ9000-TRACE1310 gas chromatography–triple quadrupole mass spectrometer was purchased from Thermo Fisher Scientific, Waltham, MA, USA. The Strata-X-A solid-phase extraction column was purchased from Supelco, Bellefonte, PA, USA. The BSA124S-CW analytical balance was purchased from Sartorius, Göttingen, Germany.

### 2.3. Test Method

#### 2.3.1. Determination of Volatile Components in *A. venetum* Honey

Zhu et al.’s [20] method with slight modifications was used. An *A. venetum* honey sample (2.0 g) was accurately weighed and placed in a 10 mL headspace bottle to which 0.2 g sodium chloride and 2 mL ultrapure water were added. Following shaking and mixing, 20 μL of 100 mg/L cyclohexanone internal standard solution was added to the mixture and the bottle mouth was immediately sealed. The sample was placed on an automatic sampler platform, and the extraction parameters were set as follows: equilibrium temperature, 45 °C; equilibrium time, 20 min; and adsorption time, 45 min.

We used a 50/30-μm divinylbenzene/carboxen/polydimethyl siloxane (DVB/CAR/PDMS) three-phase extraction head from Supelco. DVB exhibits good affinity for polar compounds. CAR is highly capable of capturing small, low-molecular-weight volatile molecules. PDMS is a nonpolar coating that can effectively adsorb nonpolar and weakly polar compounds. The combination of these compounds enables the fiber head to efficiently adsorb both polar and nonpolar compounds; therefore, it is highly suitable for the analysis of complex volatile components in a system.

The chromatographic conditions were as follows: L × I.D. 30 m × 0.25 mm, df 0.25 μm (TG-5SIMS); carrier gas, helium; flow rate, 1.0 mL/min; inlet temperature, 260 °C; injection method, splitless injection; initial temperature, 40 °C; solution delay time, 5 min; and total time, 41 min. The mass spectrometry conditions were as follows: electron energy, 70 eV; transmission line temperature, 250 °C; ion temperature, 230 °C; full-scan mode; and scan range, 45–550 Aum. Volatile components were identified by matching their mass spectral data against the National Institute of Standards and Technology (NIST) 20 mass spectral library.

#### 2.3.2. Method Validation

According to the signal-to-noise ratio of the workstation, the limit of detection (LOD) was three times the signal-to-noise ratio, and the limit of quantitation was three times the LOD. Detailed data are provided in Supplementary Table S1.

#### 2.3.3. Aroma Analysis of *A. venetum* Honey

The aroma of *A. venetum* honey was analyzed using odor activity value (OAV) and odor contribution rate (OCR). OAV is used to quantify the contribution of volatile components to honey aroma, which is defined as the ratio of the concentration of volatile components to their odor threshold (OT) in water [21,22]. While analyzing the aroma of *A. venetum* honey, volatile components with an OAV of >1 were identified as key aroma substances, and their values were positively correlated with the OAVs of the volatile components, which determined the intensity of their contribution to the aroma of the honey. Volatile components with OAVs between 0.1 and 1 are classified as modified aroma substances and play a pivotal role in the modification and optimization of the aroma characteristics of honey through synergistic interactions [23]. OCR is the ratio of the OAV of volatile components to the total OAV [24]. The higher the OAV and OCR, the greater the contribution of the substance to *A. venetum* honey, making it a key aroma substance.

The concentration of aroma compounds was calculated based on internal standards using the equation shown below.

$$C_{i}=\frac{A_{unkonwn}}{A_{IS}}\times C_{IS},$$

where *C*_i_ represents the relative concentration of the target compound (μg/kg), *A_unkonwn_* and *A_IS_* represent the peak areas of the target compound and internal standard (cyclohexanone), respectively, and *C_IS_* represents the known concentration of the internal standard.

The *OAV* of each volatile component was calculated based on its concentration as shown below.

$${OAV}_{i}=\frac{C_{i}}{{\mathrm{OT}}_{i}},$$

where *C*_i_ represents the concentration of the compound (μg/kg) and *C*_i_ represents its OT in water. OT was determined based on the findings from previous studies [25,26,27,28,29,30,31]. Components with *OAV*_i_ of ≥1 were considered significant contributors to the overall aroma profile of the honey.

The *OCR* of each volatile component was calculated based on its *OAV* as shown below.

$${OCR}_{i}=\frac{{OAV}_{i}}{{OAV}_{total}}$$

where *OAV*_i_ represents the *OAV* of the target compound and *OAV_total_* represents the total *OAV*.

#### 2.3.4. Data Processing

All experiments were conducted in triplicate, and the results are expressed as mean values with standard deviations (n = 3). Origin 2024 software was used for drawing. After standardizing the data using Graph Pad Prism 8.0.2, the data were subjected to PCA and orthogonal partial least squares discriminant analysis (OPLS-DA) analysis using SIMCA14.1. Analysis of variance was performed using IBM SPSS Statistics 27 hypothesis. Duncan’s test was employed to analyze the significant difference, and *p* < 0.05 was considered to indicate statistical significance.

## 3. Results and Analysis

### 3.1. Analysis of the Volatile Components of A. venetum Honey

**Figure 1:**
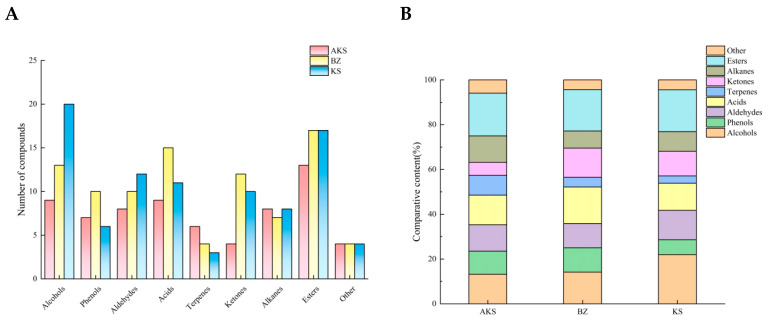
Composition of volatile components in *A. venetum* honey (number of volatile components (**A**) and relative content of volatile components (**B**)).

HS–SPME–GC–MS was employed to comprehensively characterize the volatile profile of *A. venetum* honey. Overall, 160 volatile components were identified, including 26 alcohols, 17 phenols, 16 aldehydes, 22 acids, 12 terpenes, 20 ketones, 12 alkanes, 24 esters, and 11 other substances. The composition and relative content of the volatile components in *A. venetum* honey notably differed by region (Figure 1). In honey sourced from AK and BZ, esters formed the highest proportion of volatile components, whereas the one obtained from KS showed the highest proportion of alcohols.

**Figure 2:**
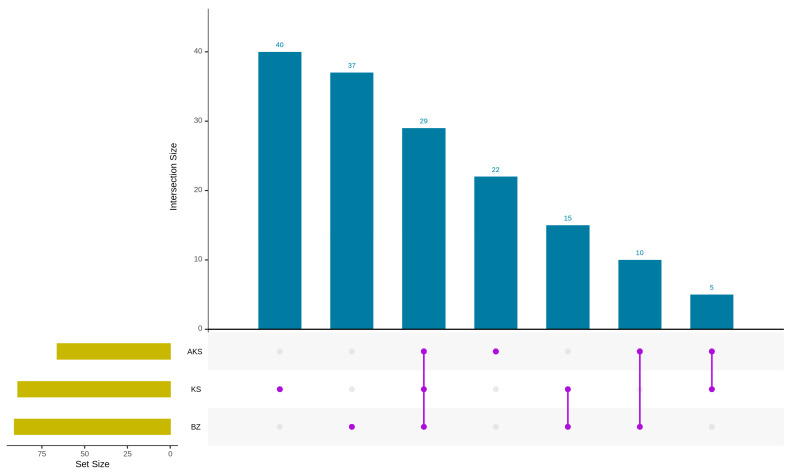
Upset diagram of the volatile components of *A. venetum* honey from different producing areas. Blue represents the data size of each intersection. Yellow represents the data size of each original set. Purple represents the intersections of different sets, denoted by different filling patterns.

Based on the UpSet diagram (Figure 2), the three honey samples share 28 common volatile components, including benzyl alcohol, benzaldehyde, decanal, and benzeneacetaldehyde. By contrast, the honey samples sourced from AKS, BZ, and KS contain 22, 37, and 40 unique components, respectively. Pairwise overlaps revealed 10 common components between the honey obtained from AKS and BZ, 5 between that obtained from AKS and KS, and 15 between that obtained from KS and BZ. The presence of shared components reflects compositional similarity between the different honey samples, likely due to them being *A. venetum* honey [32]. However, notable differences in the content of common components suggest that geographical origin can influence the volatile profile of honey, even within the same type [33].

Preharvest factors such as botanical origin, geographical conditions, and bee colony traits significantly affect the composition and quality of honey [34]. The volatile profile of *A. venetum* honey from Xinjiang likely varies with the regional environment. A study on *Artemisia* [35] showed that high altitudes promoted the accumulation of some volatile monoterpenes, whereas high temperatures and drought stress reduced nectar volume [36]. Although the terrain and climate considerably differ among AKS, KS, and BZ, ranging from mountainous and high-altitude regions to arid deserts and flat plains, the specific mechanisms through which these geographic and climatic factors influence the volatile composition of *A. venetum* honey remain unclear and warrant further investigation [37,38].

Alcohol is an important volatile component of honey, which mainly comes from its oxidative degradation of aldehydes or lipids in bees and the environment, resulting in a unique floral and fruity aroma [39]. Twenty-six types of alcohol compounds were detected in all three *A. venetum* honey samples. Of them, the KS samples exhibited the highest content, accounting for 22.22% of its total volatile content. The AKS samples exhibited the lowest alcohol content, accounting for only 13.23% of its total volatile content. 1-Nonanol imparts a citrus flavor to honey, and it was detected only in the AKS samples. Benzyl alcohol imparts a floral fragrance to honey. Although benzyl alcohol was detected in all the three samples, its content in the KS samples was significantly higher than that in the other samples.

Phenolic compounds are important aroma components. Seventeen phenolic compounds were detected in all the *A. venetum* honey samples at a relative content of 6.67–10.989%. (3R,3aR,3bR,4S,7R,7aR)-4-Isopropyl-3,7-dimethyloctahydro-1H-cyclopenta[1,3]cyclopropa[1,2]benzen-3-ol imparts a fruity aroma to honey, and this compound was detected only in the AKS samples. 2-Methoxy-4-vinylphenol imparts a floral aroma to honey, and ethanol, 2-(2-butoxyethox)-, acetate imparts an herb-like aroma; these compounds were not detected in the KS samples. Notably, the BZ samples contained 1-heptatriacotanol, which exhibits a floral aroma.

Aldehydes comprise the primary category of volatile components in honey, accounting for >30% of the volatile components. Aldehydes impart honey with a fruity or sharp fruit flavor [40]. Herein, 16 aldehydes were detected, including 8 in the AKS samples, 10 in the BZ samples, and 12 in the KS samples. Among the three samples, the KS samples exhibited the highest aldehyde content, accounting for 13.33% of their total volatile content, whereas the BZ samples exhibited the lowest content of 10.99%. The aldehyde composition of *A. venetum* honey from different areas was different. Of the 16 aldehydes, 7 were detected in all the samples: benzaldehyde; decanal; benzeneacetaldehyde; octanal; nonanal; 2-decenal, (E)-; and furfural. Dodecanal imparts a citrus flavor to honey, and only the AKS samples contained this compound.

Acids affect the flavor, aroma, and biological activities of honey. Herein, 22 acids were detected in the honey samples, with cis-5,8,11,14,17-eicosapentaenoic acid (1.26–4.99 μg/kg) and nonanoic acid (17.15–42.10 μg/kg) being present in all the samples. Acids were the most abundant compounds in the BZ samples, accounting for 16.484% of the total volatile content, and only six acids were detected in BZ samples. Eleven acids were detected in KS samples, whereas only nine were detected in the AKS samples. Interestingly, the percentage of acids in the total volatile content of the KS samples was lower than that in the total volatile content of the AKS samples, whereas the total volatile content in the AKS samples was lower than that in the KS samples. Phenylacetic acid is responsible for the characteristic “honey” flavor, and it is important that the AKS area samples contain it.

Terpenes form the core group of volatile components in honey. The terpene content of honey differs depending on nectar source and geographical region, which determines the unique flavor of honey [41]. Herein, 12 terpenes were detected, with a relative content of 3.33%–8.823%. No common terpenes were found in the three groups of samples. When compared with other substances, the content of terpenes was the lowest, and only one terpene contributing to the aroma components of honey was detected. The terpene α-pinene (1.28 μg/kg) was detected only in the BZ samples.

Ketones are important compounds in honey as they provide characteristic odor to it and contribute to its low threshold value. Ketones such as β-damascenone are key contributors to floral and fruity aromas in various honeys [42]. The content of ketones detected in this study accounted for 5.882%–13.187% of the total volatile content. Cyclohexanone (5.07–5.15 μg/kg) and 2-buten-1-one, 1-(2,6,6-trimethyl-1,3-cyclohexadien-1-yl)-, (E)- (6.01–74.74 μg/kg) were the common ketones found in all the *A. venetum* honey samples. 3H-Cyclodeca[b]furan-2-one, 4,9-dihydroxy-6-methyl-3,10-dimethylene-3a,4,7,8,9,10,11,11a-octahydro- and 5,9-undecadien-2-one,6,10-dimethyl-, (E)- were unique to the AKS samples, and eight unique compounds were detected in the BZ samples. Only six compounds were detected in the KS samples, of which only 2(3H)-furanone, 5-heptyldihydro- contributed to the honey odor.

Alkanes are common volatile components in honey. Twelve alkanes were detected in the three honey samples from Xinjiang. The relative content of alkanes in the AKS samples was the highest, accounting for 12.765% of the total volatile content. Among the alkanes, benzene, hexamethyl- (14.06 μg/kg) exhibited the highest content. Four alkanes were codetected in all the samples. The threshold of alkane compounds is generally high, and their contribution to honey flavor is small. However, under certain conditions, some alkanes can be converted into aroma compounds. Some scholars believe that alkanes are derived from beeswax and that the presence of alkanes in honey is possibly caused by beeswax residues. A research study reported that it is common to detect numerous alkanes in honey [43]. For example, 52 types of alkanes were detected in *Toddalia asiatica* honey, which accounts for 30% of its total volatile content [44].

Esters, an important class of volatile components in honey, impart a characteristic fruity flavor. A previous study [45] determined the OTs and OAVs for esters such as ethyl acetate and 1-propyl-2-methyl acetate, confirming their significant contribution to the fruity aroma. Overall, 24 esters were identified in all the honey samples. After alcohols, esters were the second most abundant volatile group, accounting for 17.582%–19.118% of the total volatile content. They constituted the predominant volatiles in the AKS and BZ samples. Among the seven esters detected, four were identified as aroma-active: ethyl octadecanoate, ethyl oleate, methyl salicylate, and ethyl nonanoate. Dodecyl formate was exclusively found in the AKS samples. The esters unique to the BZ samples included ethyl phenylacetate, ethyl hexanoate, ethyl 9-hexadecenoate, and 2-ethylhexyl 3-(4-methoxyphenyl)propenoate. Additionally, seven esters were specific to the KS samples.

### 3.2. Difference Analysis of Volatile Components in A. venetum Honey

#### 3.2.1. Systematic Cluster Analysis

**Figure 3:**
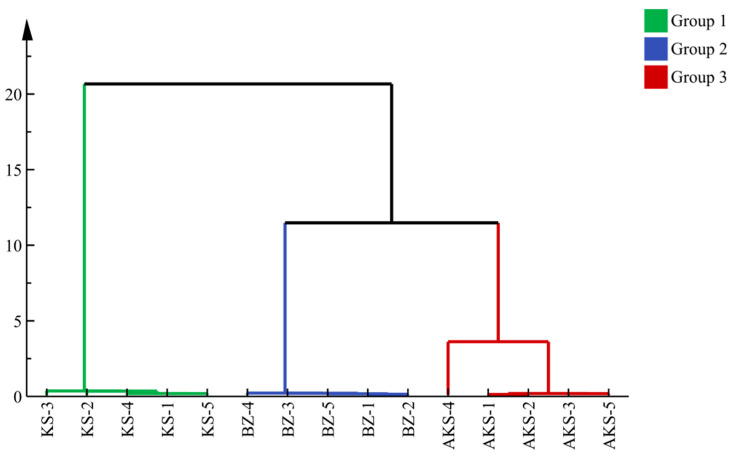
Cluster analysis of volatile substance content of *A. venetum* honey. AKS1-5 (Aksu), KA1-5 (Kashgar), and BZ1-5 (Bayinguoleng Mongolian Autonomous Prefecture).

To comprehensively understand the differences in the aroma components of the *A. venetum* honey samples obtained from three regions in Xinjiang, the detected volatile content was subjected to cluster analysis. Results were presented on a tree diagram, which provides a more intuitive representation of the relative similarity and grouping between samples. The results provide a specific sample grouping scheme to objectively reveal the natural affinity between samples. The results are presented in Figure 3. At a distance of 10, the samples can be divided into three categories: KS, BZ, and AKS samples. There were differences in the odor type and intensity among the same type of honey sourced from different regions, consistent with existing research results [46].

#### 3.2.2. PCA

**Figure 4:**
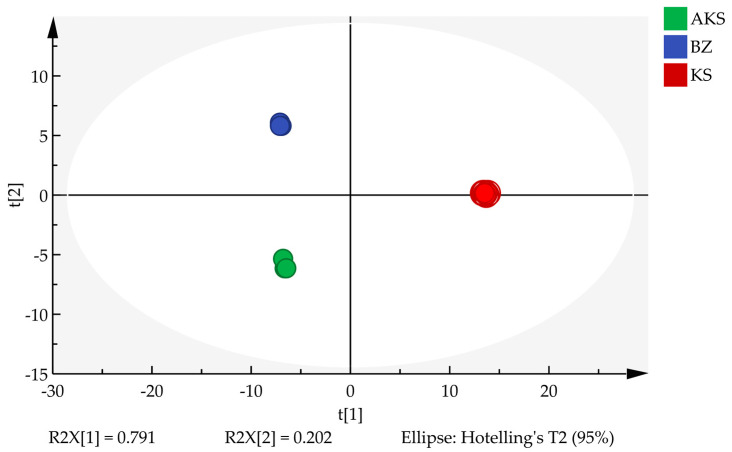
PCA scatter plot of *A. venetum* honey samples from AK (Aksu), KA (Kashgar), and BZ (Bayinguoleng Mongolian Autonomous Prefecture).

PCA is an unsupervised dimensionality reduction technique primarily used to visualize the overall structure of a dataset, reveal natural clustering trends, and identify potential outliers without relying on sample category labels. Herein, the contents of 160 volatile components were used as variables. Using SIMCA (version 14.1) software, three principal components with eigenvalues of >1 were extracted, collectively explaining 99.425% of the total variance. The exceptionally high cumulative explained variance—with the first two principal components alone accounting for 99.3%—likely reflects the high homogeneity of the volatile composition of *A. venetum* honey across the three regions and the presence of several dominant compounds that strongly contribute to the variance. These results indicate that most of the variability in the dataset was effectively captured by a small number of components, highlighting the strong structure of the data. As shown in Figure 4, all the honey samples were naturally clustered into three distinct groups, consistent with the results of cluster analysis. This clear separation suggests meaningful differences in the volatile profiles among the honey sourced from the three regions.

#### 3.2.3. OPLS-DA

PCA provides an unbiased overview and assists in quality-control analysis of a dataset, whereas OPLS-DA builds on this foundation by enabling targeted exploration based on specific hypotheses to identify statistically significant differential markers. The combination of these two approaches ensures that the analysis is both comprehensive and hypothesis-driven.

**Figure 5:**
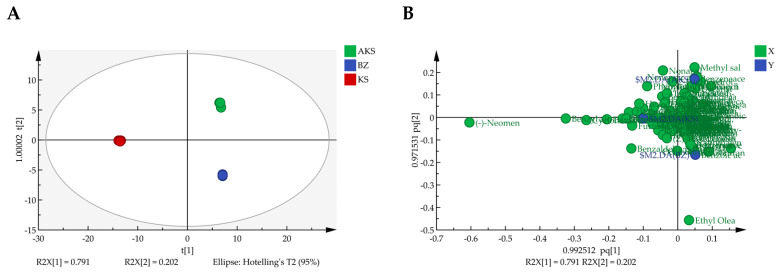
*A. venetum* honey OPLS-DA analysis diagram: (**A**) sample score plot; (**B**) load plot of *A. venetum* honey. AKS (Aksu), KA (Kashgar), and BZ (Bayinguoleng Mongolian Autonomous Prefecture).

As illustrated in Figure 5A, the *A. venetum* honey samples from the three regions formed three distinct clusters, again aligning with the clustering results. Figure 5B displays the distribution of the sample groups and volatile components in the loading plot, enabling the visual assessment of the contribution of each component to different sample groups. From a chemical and biological perspective, phenylacetic acid and nonanal exhibited the strongest correlation with the AKS samples, indicating that these compounds may be key markers for defining the characteristic floral and fruity aroma of the AKS *A. venetum* honey. Similarly, benzofuran and caprylic acid were closely linked to the BZ samples, potentially leading to the formation of regionally unique fruity flavors. At the same time, 3-cyclohexene-1-acetaldehyde, α,4-dimethyl- and other substances are highly representative in the KS samples, indicating their potential role in shaping the unique nutty and caramel-like flavors of honey sourced from this region. These findings not only highlight the statistical distinctions in the volatile and aroma profiles of the honey sourced from the three regions but also provide insights into the chemical basis underlying the geographical authentication of *A. venetum* honey.

**Figure 6:**
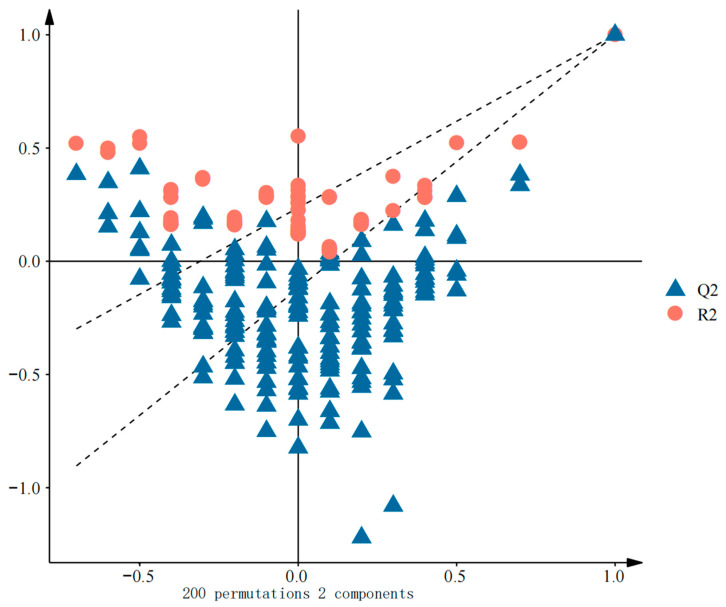
OPLS-DA model replacement test.

To assess potential overfitting of the OPLS-DA model, key parameters including R2Y and Q2 were evaluated (Figure 6). In the *A. venetum* honey model, both R2Y and Q2 reached 1.0, with all R2 and Q2 values exceeding 0.5, indicating excellent model fit and predictive capability.

Herein, R2 (R2Y) quantifies the proportion of variance explained by the model, reflecting its goodness of fit, while Q2 represents the predictive ability of the model estimated through cross-validation. A Q2 of >0.5 generally confirms robust predictability. To further validate the model, a permutation test was conducted by randomly shuffling class labels multiple times. All permuted models yielded lower R2 and Q2 values than the original model, statistically supporting the absence of overfitting and the reliability of predictions.

Regarding sample size, the distinct grouping and strong validation metrics suggest that the number of samples included in this study adequately supports model robustness. However, the generalizability of the model may be influenced by limited sample diversity or inherent biological variability. Future studies with larger sample sizes are warranted to verify the practical applicability of the model.

**Figure 7:**
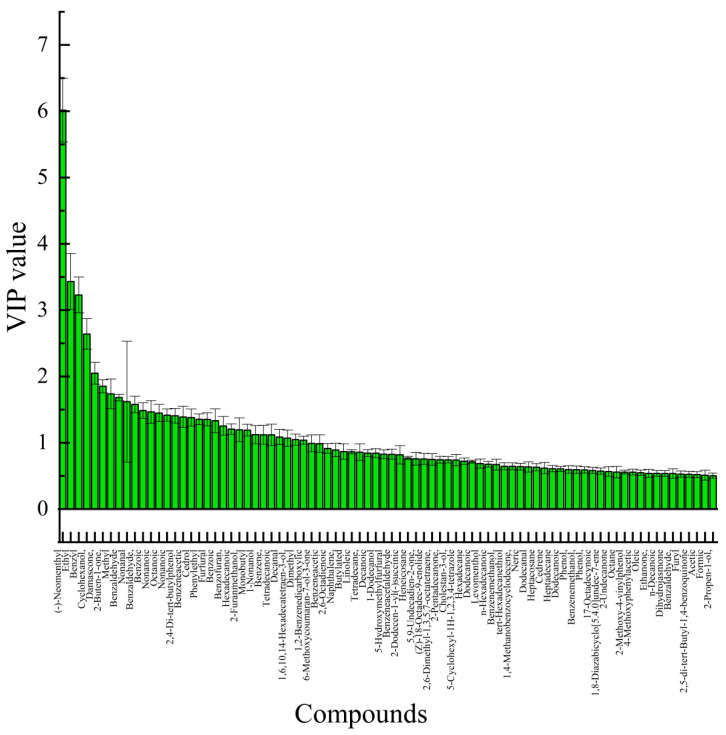
VIP diagram of the *A. venetum* honey OPLS-DA model.

Key markers were selected based on their variable importance in projection (VIP) scores from the OPLS-DA model. The VIP score quantifies the contribution of a volatile compound to the separation of geographical origins. A VIP value of >1.0 is widely adopted as a criterion for significance, as it identifies compounds with an above-average influence on the model’s discriminatory power [47]. As presented in Figure 7, 31 volatile compounds met this criterion (VIP > 1) and were thus identified as the key markers differentiating the honey sourced from the different producing areas.

Among the 31 key volatile compounds, several compounds are known to influence the aroma and quality of honey. For example, ethyl oleate, nonanal, and cyclohexanone, which had high VIP scores, are commonly associated with fruity, rosy, and peppermint-like aromas in honey, respectively. These compounds not only contribute to the distinctive aroma profile of *A. venetum* honey but also serve as potential markers for geographical authentication. Further investigation of the odor activity and synergistic effects of these volatile compounds can enhance the understanding of their role in shaping the sensory characteristics and regional identity of honey.

#### 3.2.4. Clustering Heatmap Analysis

**Figure 8:**
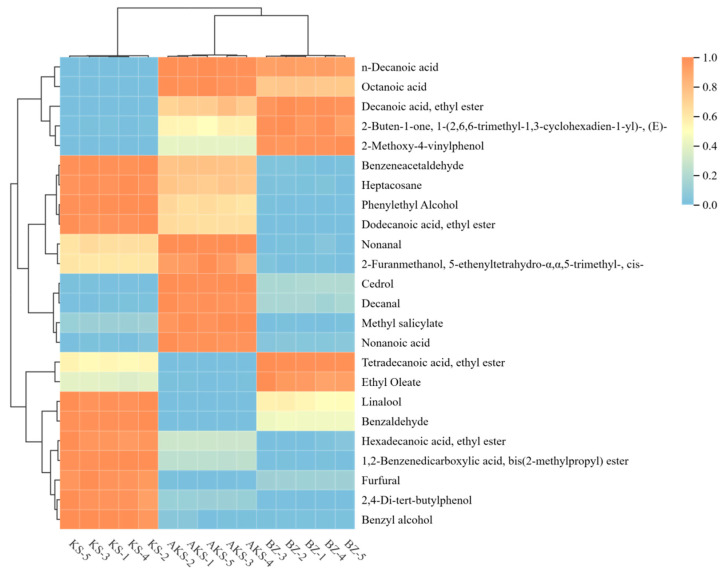
Heatmap of 16 volatile components in *A. venetum* honey samples from AKS1-5 (Aksu), KA1-5 (Kashgar), and BZ1-5 (Bayinguoleng Mongolian Autonomous Prefecture).

To better identify and differentiate the key volatile components of *A. venetum* honey sourced from the different producing areas, Duncan’s one-way analysis of variance test was combined with VIP analysis to screen out 24 different compounds with a VIP value of >1 and *p* < 0.05. After standardizing the content of the same compound contained in the samples, a cluster heat map was drawn to visually demonstrate the content differences of these compounds in the different samples. As presented in Figure 8, the main volatile components of the three *A. venetum* honey samples were different. The contents of linalool; benzaldehyde; hexadecanoic acid, ethyl ester; 1.2-benzenediearboxylic acid, bis(2-methylpropyl) ester; furfaral; 2.4-di-tert-butylphenol; and benzyl alcohol in the KS samples were considerably higher than those in the other samples. The contents of cedrol, decanal, methyl salicylate, and nonanoic acid in the AKS samples were remarkably higher than those in the other samples. The contents of phenylethyl alcohol, nonanal, and 2-furanmethanol, 5-ethenyltetrahydro-α,α,5-trimethyl-, cis- in the BZ samples were significantly lower than those in the other samples; however, the contents of 2-buten-1-one, 1-(2,6,6-trimethyl-1.3-cyclohexadien-1-y)-, (E)-; tetradecanoic acid, ethyl ester, and ethyl oleate were higher than those in the other samples.

### 3.3. Identification of Aroma Active Substances in A. venetum Honey

Thirty-eight volatile compounds with established aroma thresholds were identified in *A. venetum* honey (Supplementary Table S2). The selection of these compounds was based on two key steps: first, all compounds were initially detected through GC–MS analysis to ensure their actual presence in the honey samples; second, a filtering process was performed using aroma threshold data from the published literature, with only the compounds having clearly reported and widely recognized aroma thresholds included in the final list. Among these 38 compounds, 34 exhibited OAVs of >1 (Table 1), indicating their significant contribution to the overall aroma profile of *A. venetum* honey.

2-Buten-1-one, 1-(2,6,6-trimethyl-1,3-cyclohexadien-1-yl)-, (E)- was a key aroma compound present in all the samples. With a concentration of 6.01–74.74 μg/kg, its extremely low OT of 0.002 μg/kg led to a high OAV (3005–37370) and OCR (19.296–75.280%), contributing apple, rose, and sweet notes. Geographical differences influenced the aroma composition of the honey. AKS and BZ samples featured β-damascenone, while the dominant compound in the KS samples was 2,4-di-tert-butylphenol (OAV: 5176, OCR: 44.265%), imparting floral and almond notes. Temperature, precipitation, sunshine duration, and altitude can affect metabolic pathways in plants and alter the content of volatile precursors in their nectar [11]. Notably, the AKS samples contained a cyclopropanobenzene derivative ((3R, 3aR, 3bR, 4S, 7R, 7aR)-4-isopropyl-3,7-dimethyloctahydro-1H-cyclopenta[1,3]cyclopropa[1,2]benzen-3-ol), which had a high OAV (7000.00) and OCR (16.964%) and imparted a fruity aroma. Under different soil conditions, significant differences are observed in the composition of aromatic compounds produced by plants, which result in differences in the types and contents of volatile compounds in honey [48].

Being the main aroma active components of *A. venetum* honey, 2-buten-1-one, 1-(2,6,6-trimethyl-1,3-cyclohexadien-1-yl)-; 2,4-di-tert-butylphenol; and (3R, 3aR, 3bR, 4S, 7R,7aR)-4-isopropyl-3.7-dimethyloctahydro-1H-cyclopenta[1,3]cyclopropa[1,2]benzen-3-ol were present in the other honey samples. Damascenone is a characteristic marker of Turkish pine honey. It has the highest OAV among the aroma compounds in *T. asiatica* honey [46].

**Figure 9:**
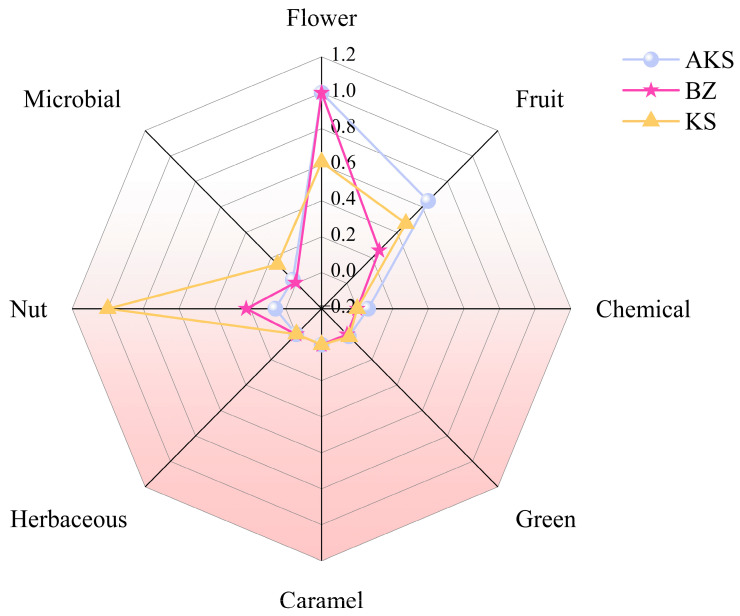
Radar map of aroma characteristics of *A. venetum* honey.

The classification of the identified aroma compounds into the eight sensory categories (flower, fruit, chemical, green, caramel, herbaceous, nut, and microbial aromas) was directly adopted from a study by Kortesniemi et al. [49], who categorized a wide range of volatile compounds based on professional sensory panel evaluations. Owing to the complexity of aroma components, some substances belong to multiple categories. As shown in Figure 9, the aroma characteristics of *A. venetum* honey sourced from different regions were different. Owing to the high content of 2-buten-1-one, 1-(2,6,6-trimethyl-1,3-cyclohexadien-1-yl)- in the AKS and BZ samples, these samples exhibited strong floral and fruity aromas. In general, the AKS samples were mainly characterized by floral, fruity, and green aromas, which were complemented by a rich herbal flavor. The BZ samples exhibited obvious floral, green, and herbaceous aromas, whereas its fruity, caramel, and nutty flavors were relatively poor. The KS samples exhibited obvious characteristics of caramel and nutty flavors. The volatile components and contents of the same type of honey from different producing areas were different. Thus, *A. venetum* honey sourced from different areas develops unique flavor attributes. The flavor profiles of *A. venetum* honey from AKS and BZ show slight similarities. Furthermore, these honey types are mainly floral and fruity, whereas honey sourced from KS is dominated by a nutty flavor, supplemented by floral and fruity flavors.

The observed aroma differentiation not only highlights the impact of geographical origin on honey flavor but also suggests potential applications in geographical traceability and quality authentication. Furthermore, these distinctive sensory profiles can be used for targeted marketing strategies and value-added product positioning, helping consumers better identify and appreciate the regional characteristics of honey.

## 4. Conclusions

This study characterized the volatile profiles of *A. venetum* honey from three regions in Xinjiang using HS-SPME–GC–MS. Overall, 160 volatile compounds were identified, with 28 compounds being common across all the regions. Chemometric analysis revealed region-specific markers: honey samples from AKS contained (3R,3aR,3bR,4S,7R,7aR)-4-isopropyl-3,7-dimethyloctahydro-1H-cyclopenta[1,3]cyclopropa[1,2]benzen-3-ol and other compounds; those obtained from BZ contained α-pinene and other compounds; and those obtained from KS contained 5-heptyldihydro-2(3H)-furanone and other compounds. OAV and OCR analyses identified 38 flavor-active compounds, which collectively shaped the distinct aroma profiles of the honey samples: AKS honey exhibited dominant floral, fruity, and green notes; BZ honey showed pronounced floral and herbaceous characteristics; KS honey was distinguished by caramel and nutty attributes.

Notably, this study has certain limitations, such as a relatively small sample size, collection of samples only at a single time point, and a lack of sensory validation, which can affect the generalizability and practical relevance of the findings. However, the identified volatile markers provide a basis for the geographical authentication, quality grading, and market positioning of *A. venetum* honey. Future studies should incorporate sensory analysis, multi-season sampling, and sensory validation under commercial conditions to enhance the scientific robustness and practical applicability of these findings.

## Figures and Tables

**Table 1:** The main aroma compounds of *A. venetum* honey.

ID	Compound	Aroma Description	AKS		BZ		KS	
			OAV	OCR%	OAV	OCR%	OAV	OCR%
1	1-Hexanol, 2-ethyl-	Sweet and flower	<1	0	<1	0	-	-
2	Phenylethyl alcohol	Rosy and flower	2.708	0.007	<1	0	3.757	0.032
3	Benzyl alcohol	Flower	<1	0	<1	0	22.131	0.189
4	1-Nonanol	Citrus	<1	0	-	-	-	-
5	Cedrol	Violet, woody, fruity	61.960	0.150	20.060	0.041	10.640	0.091
6	Linalool	Flower	-	0.000	10.400	0.021	19.100	0.163
7	1-Dodecanol	Citrus	-	0.000	-	-	741.500	6.341
8	2,4-Di-tert-butylphenol	Almondy	1345.000	3.260	8440	1.727	5176.000	44.265
9	(3R,3aR,3bR,4S,7R,7aR)-4-isopropyl-3,7-dimethyloctahydro-1H-cyclopenta[1,3]cyclopropa[1,2]benzen-3-ol	Fruity	7000	16.964	-	-	-	-
10	2-Methoxy-4-vinylphenol	Flower	223.000	0.540	546.000	1.117	-	-
11	1-Heptatriacotanol	Flower		0.000	1.840	0.004	-	-
12	Ethanol, 2-(2-butoxyethoxy)-, acetate	Herbaceous	22.400	0.054	26.400	0.054	-	-
13	Benzaldehyde	Almondy	<1	0.000	1.084	0.002	2.343	0.020
14	Decanal	Mandarin orange	6.139	0.015	2.517	0.005	1.825	0.016
15	Dodecanal	Mandarin orange	906.000	2.196	-	-	-	-
16	Benzeneacetaldehyde	Flower, honey, and green	2.178	0.005	<1	0	2.535	0.022
17	Octanal	Mandarin orange, fat, and green	1.900	0.005	1.757	0.004	1.086	0.009
18	Nonanal	Fat, mandarin orange, and rosy	41.664	0.101	9.364	0.019	36.064	0.308
19	Furfural	Bread and almondy	2.647	0.006	4.380	0.009	15.873	0.136
20	phenylacetic acid	Honey	217.000	0.526	-	-	-	-
21	Nonanoic acid	Fat and cheese	14.033	0.034	6.027	0.012	5.717	0.049
22	Octanoic acid	Cheese	8.840	0.021	6.700	0.014	-	-
23	n-Decanoic acid	Fat	3.105	0.008	2.900	0.006	-	-
24	α-Pinene	Pinewood, resin, and mandarin orange	-	0.000	128.000	0.262	-	-
25	2(3H)-furanone, 5-heptyldihydro-	Flower	-	0.000	-	-	15.200	0.130
26	Cyclohexanone	Peppermint	3.433	0.008	3.380	0.007	3.387	0.029
27	2-Buten-1-one, 1-(2,6,6-trimethyl-1,3-cyclohexadien-1-yl)-, (E)-	Flower, honey, and apple	22,235.000	53.886	37,370	76.453	3005.000	25.699
28	Benzene, hexamethyl-	Drug-like	1406.000	3.407	-	-	-	-
29	Octane	Petrol fumes	1.760	0.004	-	-	<1	0
30	Heptacosane	Rotten smell	619.000	1.500	176.000	0.360	764.000	6.534
31	Phenylacetic acid, ethyl ester	Flower	-	0.000	<1	0	-	-
32	Octanoic acid, ethyl ester	Fruity	<1	0.000	<1	0	-	-
33	Dodecanoic acid, ethyl ester	Fruity	397.000	0.962	-	-	598.000	5.114
34	Decanoic acid, ethyl ester	Fruity	5865.000	14.214	7840	16.039	-	-
35	Octadecanoic acid, ethyl ester	Fruity	132.000	0.320	131.000	0.268	176.000	1.505
36	Ethyl oleate	Fruity	346.200	0.839	1689.900	3.457	882.200	7.545
37	Methyl salicylate	Dongqing oil and peppermint	398.700	0.966	57.900	0.118	97.900	0.837
38	Acetic acid, phenylmethyl ester	Flower	-	-	-	-	113.000	0.966

Note: The aroma description is referenced to an online flavor database (http://www.flavornet.org/index.html, accessed on 18 July 2025); “-“ indicates not detected.

## Data Availability

The original contributions presented in this study are included in the article/supplementary material. Further inquiries can be directed to the corresponding author.

## Supplementary Materials

The following supporting information can be downloaded at: https://www.mdpi.com/article/10.3390/foods14223860/s1, Table S1: The limit of detection and limit of quantitation of volatile components; Table S2: Volatile compounds content of *A. venetum* honey in different regions.
