# Supplementary material for: Comparison of the Volatile Components of Apocynum venetum Honey from Different Production Areas in Xinjiang
Source: Foods. 2025 Nov 11;14(22):3860. doi: 10.3390/foods14223860 (PMC12650918; doi:10.3390/foods14223860)
Supplement: Supplementary file 1 [file foods-14-03860-s001.zip › foods-3910969-supplementary.pdf]

Supplementary Material S1

ID	CAS	Compound	LOD/(μg/kg)	LOQ/(μg/kg)
1	104-76-7	1-Hexanol, 2-ethyl-	0.09	0.27
2	1960-12-8	Phenylethyl Alcohol	0.80	2.4
3	100-51-6	Benzyl alcohol	0.07	0.21
4	629-96-9	1-Eicosanol	0.15	0.45
5	5989-33-3	2-Furanmethanol, 5-ethenyltetrahydro-α,α, 5-trimethyl-, cis-	5.12	15.36
6	143-08-8	1-Nonanol	5.18	15.54
7	22599-96-8	Cholestan-3-ol, 2-methylene-, (3β,5α)-	1.59	4.77
8	1113-21-9	1,6,10,14-Hexadecatetraen-3-ol, 3,7,11,15-tetramethyl-, (E,E)-	2.58	7.74
9	1197-01-9	Benzenemethanol, α,α,4-trimethyl-	0.86	2.58
10	25360-09-2	tert-Hexadecanethiol	0.08	0.24
11	562-74-3	Terpinen-4-ol	0.36	1.08
12	56554-77-9	13-Heptadecyn-1-ol	0.18	0.54
13	77-53-2	Cedrol	1.69	5.07
14	78-70-6	Linalool	0.09	0.27
15	10198-23-9	Cyclohexanol, 1-methyl-4-(1-methylethenyl)-, acetate1-methyl-4-(1-methylethenyl)-, acetate	0.19	0.57
16	105-13-5	Benzenemethanol, 4-methoxy-	1.85	5.55
17	112-53-8	1-Dodecanol	2.39	7.17
18	1197-34-8	Phenol, 3,5-diethyl-	0.58	1.74
19	16409-4	Cyclohexanol,	5.39	16.17

	5-3	5-methyl-2-(1-methylethyl)-, acetate		
20	2216-51-5	Levomenthol	2.37	7.11
21	2490-48-4	1-Hexadecanol, 2-methyl-	1.08	3.24
22	35727-45-8	Cyclohexanol, 3-ethenyl-3-methyl-2-(1-methylethenyl)-6-(1-methylethyl)-, [1R-(1α,2α,3β,6α)]-	0.59	1.77
23	38022-97-8	Ageratriol	0.39	1.17
24	4808-01-9	2-Propen-1-ol, 3-(2,6,6-trimethyl-1-cyclohexen-1-yl)-	0.58	1.74
25	6750-34-1	1-Dodecanol, 3,7,11-trimethyl-	0.39	1.17
26	96-76-4	2,4-Di-tert-butylphenol	0.46	1.38
27	38230-60-3	(3R,3aR,3bR,4S,7R,7aR)-4-Isopropyl-3,7-dimethyloctahydro-1H-cyclopenta[1,3]cyclopropa[1,2]benzen-3-ol	0.06	0.18
28	7786-61-0	2-Methoxy-4-vinylphenol	0.56	1.68
29	10396-80-2	2,6-Di-tert-butyl-4-hydroxy-4-methylcyclohexa-2,5-dien-1-one	0.08	0.24
30	105794-58-9	1-Heptatriacotanol	0.06	0.18
31	124-17-4	Ethanol, 2-(2-butoxyethoxy)-, acetate	0.16	0.48
32	128-37-0	Butylated Hydroxytoluene	2.37	7.11
33	1941-12-4	Phenol, 2-methoxy-3-(2-propenyl)-	1.68	5.04
34	28400-11-5	β-Acorenol	0.06	0.18
35	30364-38-6	Naphthalene, 1,2-dihydro-1,1,6-trimethyl-	0.23	0.69

36	41432-70-6	β-Longipinene	0.36	1.08
37	501-92-8	Phenol, 4-(2-propenyl)-	0.51	1.53
38	514-95-4	1,5,5-Trimethyl-6-methylene-cyclohexene	0.06	0.18
39	527-54-8	Phenol, 3,4,5-trimethyl-	0.31	0.93
40	527-60-6	Phenol, 2,4,6-trimethyl-	0.05	0.15
41	62690-65-7	Naphthalene, 1,2,3,5,8,8a-hexahydro-	1.28	3.84
42	87-44-5	Caryophyllene	0.53	1.59
43	97306-66-6	5H-Benzo[b]pyran-8-ol, 2,3,5,5,8a-pentamethyl-6,7,8,8a-tetrahydro-	0.86	2.58
44	100-52-7	Benzaldehyde	1.36	4.08
45	112-31-2	Decanal	0.28	0.84
46	112-54-9	Dodecanal	0.06	0.18
47	116-26-7	1,3-Cyclohexadiene-1-carboxaldehyde, 2,6,6-trimethyl-	0.06	0.18
48	122-78-1	Benzeneacetaldehyde	1.36	4.08
49	123-11-5	Benzaldehyde, 4-methoxy-	2.34	7.02
50	124-13-0	Octanal	0.06	0.18
51	124-19-6	Nonanal	2.96	8.88
52	134-96-3	Benzaldehyde, 4-hydroxy-3,5-dimethoxy-	0.06	0.18
53	29548-14-9	3-Cyclohexene-1-acetaldehyde, α,4-dimethyl-	0.06	0.18
54	3913-81-3	2-Decenal, (E)-	0.53	1.59
55	67-47-0	5-Hydroxymethylfurfural	1.26	3.78
56	75697-98-2	2-Isopropenyl-5-methylhex-4-enal	0.02	0.06
57	823-82-	2,5-Furandicarboxaldehy	0.53	1.59

	5	de		
58	98-01-1	Furfural	0.54	1.62
59	99172-18-6	3,5-Heptadienal, 2-ethylidene-6-methyl-	0.05	0.15
60	103-82-2	Benzeneacetic acid	0.36	1.08
61	10417-94-4	cis-5,8,11,14,17-Eicosapentaenoic acid	0.05	0.15
62	112-05-0	Nonanoic acid	1.05	3.15
63	124-07-2	Octanoic acid	0.09	0.27
64	143-07-7	Dodecanoic acid	1.06	3.18
65	1443-76-1	Benzoic acid, 4-hydroxy-3,5-dimethoxy-, hydrazide	2.06	6.18
66	146502-80-9	(-)-Neomenthyl acetate	10.69	32.07
67	17735-94-3	cis-13-Eicosenoic acid	0.19	0.57
68	19780-11-1	2-Dodecen-1-yl(-)succinic anhydride	0.34	1.02
69	2613-89-0	Propanedioic acid, phenyl-	0.06	0.18
70	334-48-5	n-Decanoic acid	1.28	3.84
71	34450-18-5	17-Octadecynoic acid	1.09	3.27
72	3682-17-5	Benzenepropanoic acid, α-(hydroxyimino)-	0.08	0.24
73	4613-38-1	Neric acid	0.31	0.93
74	4726-96-9	L-Serine, O-(phenylmethyl)-	0.63	1.89
75	506-17-2	cis-Vaccenic acid	0.25	0.75
76	544-63-8	Tetradecanoic acid	0.53	1.59
77	5561-99-9	cis-11-Eicosenoic acid	0.04	0.12
78	57-10-3	n-Hexadecanoic acid	0.36	1.08
79	693-71-0	trans-13-Octadecenoic acid	0.06	0.18

80	84-69-5	1,2-Benzenedicarboxylic acid, bis(2-methylpropyl) ester	1.36	4.08
81	112-80-1	Oleic Acid	0.53	1.59
82	11028-42-5	Cedrene	0.84	2.52
83	13466-78-9	3-Carene	0.34	1.02
84	1599-67-3	1-Docosene	0.09	0.27
85	23445-02-5	(3S,3aR,3bR,4S,7R,7aR)-4-Isopropyl-3,7-dimethyloctahydro-1H-cyclopenta[1,3]cyclopropa[1,2]benzen-3-ol	0.06	0.18
86	24470-48-2	2-Pentenoic acid, 5-(decahydro-5,5,8a-trimethyl-2-methylene-1-naphthalenyl)-3-methyl-, [1S-[1α(E),4aβ,8aα]]-	0.04	0.12
87	460-01-5	2,6-Dimethyl-1,3,5,7-octatetraene, E,E-	1.03	3.09
88	6674-22-2	1,8-Diazabicyclo[5.4.0]undec-7-ene	0.08	0.24
89	7220-78-2	4,8,13-Cyclotetradecatriene-1,3-diol, 1,5,9-trimethyl-12-(1-methylethyl)-	0.36	1.08
90	74635-85-1	Uvidin C	0.26	0.78
91	74708-73-9	1,4-Methanobenzocyclodecene, 1,2,3,4,4a,5,8,9,12,12a-decahydro-	0.83	2.49
92	80-56-8	α-Pinene	0.06	0.18
93	96480-63-6	5-Hydroxy-10,10-dimethyl-6-methylenebicyclo[7.2.0]undecan-2-one	0.63	1.89
94	102488-09-5	3-Hydroxy-β-damascone	0.23	0.69
95	104-67-6	2(3H)-Furanone, 5-heptyldihydro-	0.21	0.63
96	108-94-	Cyclohexanone	0.38	1.14

	1			
97	112-12-9	2-Undecanone	1.08	3.24
98	1128-08-1	Dihydrojasmone	0.68	2.04
99	1192-62-7	Ethanone, 1-(2-furanyl)-	1.39	4.17
100	119875-15-9	3H-Cyclodeca[b]furan-2-one, 4,9-dihydroxy-6-methyl-3,10-dimethylene-3a,4,7,8,9,10,11,11a-octahydro-	0.09	0.27
101	17678-19-2	Furyl hydroxymethyl ketone	0.19	0.57
102	204648-65-7	6-Methoxycoumaran-7-ol-3-one	0.05	0.15
103	23726-91-2	Damascone, β-	0.12	0.36
104	23726-93-4	2-Buten-1-one, 1-(2,6,6-trimethyl-1,3-cyclohexadien-1-yl)-, (E)-	0.63	1.89
105	2460-77-7	2,5-di-tert-Butyl-1,4-benzoquinone	1.36	4.08
106	35044-68-9	2-Buten-1-one, 1-(2,6,6-trimethyl-1-cyclohexen-1-yl)-	0.53	1.59
107	3796-70-1	5,9-Undecadien-2-one, 6,10-dimethyl-, (E)-	0.86	2.58
108	43219-68-7	Ethanone, 1-(1,4-dimethyl-3-cyclohexen-1-yl)-	0.96	2.88
109	496-16-2	Benzofuran, 2,3-dihydro-	1.09	3.27
110	502-69-2	2-Pentadecanone, 6,10,14-trimethyl-	0.35	1.05
111	52210-15-8	2-Cyclohexen-1-one, 4-(3-hydroxy-1-butenyl)-3,5,5-trimethyl-, [R-[R*,R*-(E)]]-	0.15	0.45
112	54344-66-0	2,5,5,8a-Tetramethyl-6,7,8,8a-tetrahydro-5H-chromen-3-one	0.16	0.48
113	6280-34-8	5-Cyclohexyl-1H-1,2,3,4-tetrazole	0.21	0.63

114	87-85-4	Benzene, hexamethyl-	0.08	0.24
115	111-65-9	Octane	0.06	0.18
116	111-85-3	Octane, 1-chloro-	0.03	0.09
117	14905-56-7	Tetradecane, 2,6,10-trimethyl-	0.53	1.59
118	527-84-4	o-Cymene	0.15	0.45
119	544-76-3	Hexadecane	0.53	1.59
120	593-49-7	Heptacosane	0.26	0.78
121	629-59-4	Tetradecane	0.36	1.08
122	629-78-7	Heptadecane	0.61	1.83
123	629-92-5	Nonadecane	0.29	0.87
124	629-94-7	Heneicosane	0.49	1.47
125	630-04-6	Hentriacontane	0.51	1.53
126	101-97-3	Benzeneacetic acid, ethyl ester	1.38	4.14
127	106-32-1	Octanoic acid, ethyl ester	1.25	3.75
128	106-33-2	Dodecanoic acid, ethyl ester	0.65	1.95
129	107141-15-1	Tridecanoic acid, 3-hydroxy-, ethyl ester	0.64	1.92
130	110-38-3	Decanoic acid, ethyl ester	1.86	5.58
131	111-61-5	Octadecanoic acid, ethyl ester	0.36	1.08
132	111-62-6	Ethyl Oleate	0.19	0.57
133	119-36-8	Methyl salicylate	0.05	0.15
134	123-29-5	Nonanoic acid, ethyl ester	0.05	0.15
135	123-66-0	Hexanoic acid, ethyl ester	0.06	0.18
136	124-06-	Tetradecanoic acid, ethyl	1.06	3.18

	1	ester		
137	13058-12-3	2,6-Octadienoic acid, 3,7-dimethyl-, ethyl ester	0.53	1.59
138	131-11-3	Dimethyl phthalate	0.57	1.71
139	140-11-4	Acetic acid, phenylmethyl ester	0.63	1.89
140	14062-18-1	4-Methoxyphenylacetic acid ethyl ester	0.21	0.63
141	28303-42-6	Formic acid, dodecyl ester	0.53	1.59
142	544-35-4	Linoleic acid ethyl ester	0.57	1.71
143	54546-22-4	Ethyl 9-hexadecenoate	0.09	0.27
144	5466-77-3	2-Propenoic acid, 3-(4-methoxyphenyl)-, 2-ethylhexyl ester	0.03	0.09
145	56051-53-7	Cyclopropanebutanoic acid, 2-[[2-[[2-[(2-pentylcyclopropyl)methyl]cyclopropyl]methyl]cyclopropyl]methyl]-, methyl ester	0.05	0.15
146	606-45-1	Benzoic acid, 2-methoxy-, methyl ester	0.53	1.59
147	628-97-7	Hexadecanoic acid, ethyl ester	0.74	2.22
148	80060-76-0	(Z)-18-Octadec-9-enolide	0.68	2.04
149	93-89-0	Benzoic acid, ethyl ester	0.23	0.69
150	10152-71-3	Cyclopropaneoctanoic acid, 2-[[2-[(2-ethylcyclopropyl)methyl]cyclopropyl]methyl]-, methyl ester	1.23	3.69
151	104-93-8	Benzene, 1-methoxy-4-methyl-	2.94	8.82
152	1195-32-0	Benzene, 1-methyl-4-(1-methylethenyl)-	1.08	3.24
153	13217-66-8	2,2'-Azobis-2-methylpropanimidamide	1.06	3.18
154	13679-4	Furan, 3-phenyl-	0.05	0.15

	1-9			
155	16409-43-1	2H-Pyran, tetrahydro-4-methyl-2-(2-methyl-1-propenyl)-	0.31	0.93
156	1786-08-9	2H-Pyran, 3,6-dihydro-4-methyl-2-(2-methyl-1-propenyl)-	0.05	0.15
157	23095-44-5	Girinimbine	0.03	0.09
158	30364-38-6	Naphthalene, 1,2-dihydro-1,1,6-trimethyl-	0.12	0.36
159	30364-38-6	Naphthalene, 1,2-dihydro-1,1,6-trimethyl-	0.25	0.75
160	41678-29-9	2H-1-Benzopyran, 3,5,6,8a-tetrahydro-2,5,5,8a-tetramethyl-, trans-	0.09	0.27

Volatile compounds content of *A. venetum* honey in different regions

Note : ' - ', Not detected ; there were significant differences in the representation of different letters after the content of compounds ($p < 0.05$).

ID	CAS	RT(min)	Compound	Threshold value/(μg/kg)[25-31]	Content/(μg/kg)		
					AKS	BZ	KS
1	104-76-7	10.090	1-Hexanol, 2-ethyl-	5.4	1.01±0.08 a	1.12±0.13 a	-
2	1960-12-8	12.548	Phenylethyl Alcohol	10	27.08±5.21b	7.39±1.23 a	37.57±6.52 c
3	100-51-6	10.227	Benzyl alcohol	10	5.31±0.75 b	2.02±0.84 a	221.31±10.45 c
4	629-96-9	35.487	1-Eicosanol		3.22±0.21 b	1.83±0.53 a	-
5	5989-33-3	11.354	2-Furanmethanol, 5-ethenyltetrahydro-α,α,5-tri methyl-, cis-		30.00±4.65 c	13.71±2.78 a	24.32±8.53 b
6	143-08-8	14.299	1-Nonanol	50	14.15±1.58 a	-	-
7	22599-96-8	31.570	Cholestan-3-ol, 2-methylene-, (3β,5α)-		2.36±0.57 a	-	12.19±4.37 b
8	1113-21-9	34.357	1,6,10,14-Hexadecatetraen-3-ol, 3,7,11,15-tetramethyl-, (E,E)-		-	13.12±2.54 a	-
9	1197-01-9	14.755	Benzenemethanol, α,α,4-trimethyl-		-	3.83±0.58 a	4.84±0.36 b
10	25360-09-2	26.972	tert-Hexadecanethiol		-	0.16±0.07 a	8.77±1.32 b
11	562-74-3	14.597	Terpinen-4-ol		-	1.12±0.04 a	-
12	56554-77-9	35.041	13-Heptadecyn-1-ol		-	0.36±0.05 a	-
13	77-53-2	25.795	Cedrol	0.5	30.98±6.35 c	10.03±1.45 b	5.32±0.25 a
14	78-70-6	12.196	Linalool	0.2	-	2.08±0.15 a	3.82±0.54 b

15	10198-23-9	10.107	Cyclohexanol, 1-methyl-4-(1-methylethenyl) -, acetate1-methyl-4-(1-methylethenyl)-, acetate		-	-	3.64±0.35 a
16	105-13-5	17.458	Benzenemethanol, 4-methoxy-		-	-	9.42±2.78 a
17	112-53-8	22.455	1-Dodecanol	0.02	-	-	14.83±4.36 a
18	1197-34-8	17.877	Phenol, 3,5-diethyl-		-	-	7.29±2.37 a
19	16409-45-3	17.656	Cyclohexanol, 5-methyl-2-(1-methylethyl)-, acetate		-	-	145.34±11.25 a
20	2216-51-5	14.493	Levomenthol		-	-	9.84±2.36 a
21	2490-48-4	27.230	1-Hexadecanol, 2-methyl-		-	-	5.13±1.25 a
22	35727-45-8	21.448	Cyclohexanol, 3-ethenyl-3-methyl-2-(1-methylethenyl)-6-(1-methylethyl)-, [1R-(1α,2α,3β,6α)]-		-	-	2.12±0.45 a
23	38022-97-8	22.854	Ageratriol		-	-	2.07±0.36 a
24	4808-01-9	22.072	2-Propen-1-ol, 3-(2,6,6-trimethyl-1-cyclohexen-1-yl)-		-	-	5.27±0.14a
25	6750-34-1	31.549	1-Dodecanol, 3,7,11-trimethyl-		-	-	3.64±2.36 a
26	96-76-4	23.236	2,4-Di-tert-butylphenol	0.01	13.45±1.89 b	8.44±1.73 a	51.76±10.61 c
Total content of					127.56±20.37 b	65.21±10.14 a	578.49±86.13 c

alcohols							
27	38230-60-3	23.618	(3R,3aR,3bR,4S,7R,7aR)-4-Isopropyl-3,7-dimethyloctahydro-1H-cyclopenta[1,3]cyclopropa[1,2]benzen-3-ol	0.1	0.70±0.02 a	-	-
28	7786-61-0	18.186	2-Methoxy-4-vinylphenol	0.01	2.23±0.47 a	5.46±1.29 b	-
29	10396-80-2	22.079	2,6-Di-tert-butyl-4-hydroxy-4-methylcyclohexa-2,5-dien-1-one		1.65±0.13 a	-	-
30	105794-58-9	25.020	1-Heptatriacotanol	0.25	-	0.46±0.07 a	-
31	124-17-4	19.550	Ethanol, 2-(2-butoxyethoxy)-, acetate	0.05	1.12±0.05 a	1.320±0.12 a	-
32	128-37-0	23.189	Butylated Hydroxytoluene		8.50±1.57 b	-	1.15±0.08 a
33	1941-12-4	19.715	Phenol, 2-methoxy-3-(2-propenyl)-		2.06±0.35 a	3.59±1.76 c	2.62±0.15 b
34	28400-11-5	21.177	β-Acorenol		-	0.750±0.04 a	-
35	30364-38-6	19.396	Naphthalene, 1,2-dihydro-1,1,6-trimethyl-		-	-	3.94±1.38 a
36	41432-70-6	20.932	β-Longipinene		-	-	1.57±0.09 a
37	501-92-8	16.566	Phenol, 4-(2-propenyl)-		-	4.060±1.57 a	-
38	514-95-4	9.751	1,5,5-Trimethyl-6-methylene-cyclohexene		-	0.260±0.07 a	2.02±0.09 b
39	527-54-8	18.310	Phenol, 3,4,5-trimethyl-		1.61±0.31 a	-	-
40	527-60-6	18.313	Phenol, 2,4,6-trimethyl-		-	0.840±0.07 a	-
41	62690-65-7	22.086	Naphthalene, 1,2,3,5,8,8a-hexahydro-		-	9.110±2.54 a	-

42	87-44-5	21.170	Caryophyllene		-	-	3.52±0.31 a
43	97306-66-6	22.149	5H-Benzo[b]pyran-8-ol, 2,3,5,5,8a-pentamethyl-6,7,8,8a-tetrahydro-		-	2.080±0.07 a	-
Total content of phenols					17.870±4.36 b	27.930±8.94 c	14.820±6.75 a
44	100-52-7	26.660	Benzaldehyde	24	1.44±0.04 a	26.02±6.41 b	56.24±9.71 c
45	112-31-2	15.288	Decanal	3.6	22.10±5.75 c	9.06±4.37 b	6.57±2.04 a
46	112-54-9	20.818	Dodecanal	0.005	4.53±1.38 a	-	-
47	116-26-7	15.114	1,3-Cyclohexadiene-1-carboxaldehyde, 2,6,6-trimethyl-		-	1.06±0.07 a	-
48	122-78-1	10.499	Benzeneacetaldehyde	6.3	13.72±4.36 b	6.07±2.81 a	15.97±6.37 c
49	123-11-5	16.653	Benzaldehyde, 4-methoxy-		-	-	51.97±8.37 a
50	124-13-0	9.312	Octanal	0.7	1.33±0.14 b	1.23±0.31 b	0.76±0.08 a
51	124-19-6	12.313	Nonanal	1.1	45.83±5.37 c	10.30±4.31 a	39.67±5.36 b
52	134-96-3	26.660	Benzaldehyde, 4-hydroxy-3,5-dimethoxy-		-	3.29±0.80 a	-
53	29548-14-9	15.684	3-Cyclohexene-1-acetaldehyde, α,4-dimethyl-		-	-	0.38±0.04 a
54	3913-81-3	16.857	2-Decenal, (E)-		2.43±0.36 b	1.37±0.73 a	3.25±1.21 c
55	67-47-0	15.757	5-Hydroxymethylfurfaral		-	-	14.28±2.38 a
56	75697-98-2	14.537	2-Isopropenyl-5-methylhex-4-enal		-	1.98±0.09 a	-
57	823-82-5	11.448	2,5-Furandicarboxaldehyde		-	-	2.71±0.03 a
58	98-01-1	4.801	Furfural	3	7.94±1.37 a	13.14±2.84 b	47.62±9.51 c
59	99172-18-6	17.572	3,5-Heptadienal,		-	-	1.02±0.04 a

			2-ethylidene-6-methyl-				
Total content of aldehydes					99.32±25.75 b	73.52±21.10 a	240.44±43.12 c
60	103-82-2	16.529	Benzeneacetic acid	0.1	21.7±1.57 a	-	-
61	10417-94-4	36.459	cis-5,8,11,14,17-Eicosapentae noic acid		1.26±0.04 a	3.44±1.24 b	4.99±2.07 c
62	112-05-0	17.079	Nonanoic acid	3	42.10±9.25 b	18.08±0.72 a	17.15±2.74 a
63	124-07-2	14.409	Octanoic acid	0.5	4.42±1.74 b	3.35±0.57 a	-
64	143-07-7	24.557	Dodecanoic acid		-	4.44±1.37 a	9.92±2.67 b
65	1443-76-1	29.122	Benzoic acid, 4-hydroxy-3,5-dimethoxy-, hydrazide		-	20.37±6.61 a	-
66	146502-80-9	17.696	(-)-Neomenthyl acetate		-	-	755.15±53.74 a
67	17735-94-3	40.403	cis-13-Eicosenoic acid		-	0.74±0.08 a	-
68	19780-11-1	31.838	2-Dodecen-1-yl(-)succinic anhydride		7.59±1.37 b	-	1.32±0.08 a
69	2613-89-0	16.401	Propanedioic acid, phenyl-		-	0.84±0.06 a	-
70	334-48-5	19.664	n-Decanoic acid	2	6.21±1.27 b	5.80±2.74 a	-
71	34450-18-5	18.115	17-Octadecynoic acid		-	-	7.08±2.37 b
72	3682-17-5	13.263	Benzenepropanoic acid, α-(hydroxyimino)-		0.99±0.17 a	-	-
73	4613-38-1	19.238	Neric acid		-	-	8.54±2.74 a
74	4726-96-9	10.600	L-Serine, O-(phenylmethyl)-		-	-	3.43±1.03 a
75	506-17-2	30.922	cis-Vaccenic acid		-	1.16±0.13 a	-
76	544-63-8	29.024	Tetradecanoic acid		-	1.31±0.05 a	-
77	5561-99-9	31.456	cis-11-Eicosenoic acid		-	0.38±0.03 a	0.84±0.07 b

78	57-10-3	33.119	n-Hexadecanoic acid	7.36±2.13 b	2.60±0.17 a	-
79	693-71-0	35.762	trans-13-Octadecenoic acid	-	0.43±0.04 a	-
80	84-69-5	31.083	1,2-Benzenedicarboxylic acid, bis(2-methylpropyl) ester	12.54±4.31 b	6.86±1.27 a	30.56±8.31 c
81	112-80-1	33.247	Oleic Acid	-	1.03±0.04 a	6.53±1.03 b
Total content of acids				104.17±45.31 b	70.83±12.74 a	845.51±86.74 c
82	11028-42-5	21.100	Cedrene	4.35±2.17 a	-	-
83	13466-78-9	10.979	3-Carene	-	1.20±0.04 a	-
84	1599-67-3	35.500	1-Docosene	-	2.48±1.03 a	2.37±0.45 a
85	23445-02-5	23.612	(3S,3aR,3bR,4S,7R,7aR)-4-Isopropyl-3,7-dimethyloctahydro-1H-cyclopenta[1,3]cyclopropa[1,2]benzen-3-ol	0.59±0.04 a	-	-
86	24470-48-2	32.509	2-Pentenoic acid, 5-(decahydro-5,5,8a-trimethyl-2-methylene-1-naphthalenyl)-3-methyl-, [1S-[1α(E),4aβ,8aα]]-	0.40±0.06 a	-	-
87	460-01-5	13.400	2,6-Dimethyl-1,3,5,7-octatetraene, E,E-	-	6.42±1.47 a	-
88	6674-22-2	5.294	1,8-Diazabicyclo[5.4.0]undec-7-ene	-	-	6.86±1.38 a
89	7220-78-2	30.658	4,8,13-Cyclotetradecatriene-1,3-diol, 1,5,9-trimethyl-12-(1-methyle	2.02±0.27 a	-	-

			thyl)-				
90	74635-85-1	25.714	Uvidin C		1.04±0.08 a	-	-
91	74708-73-9	26.402	1,4-Methanobenzocyclodecene, 1,2,3,4,4a,5,8,9,12,12a-decahydro-		-	-	8.66±2.41 a
92	80-56-8	7.374	α-Pinene	0.01	-	1.28±0.25 a	-
93	96480-63-6	26.113	5-Hydroxy-10,10-dimethyl-6-methylenebicyclo[7.2.0]undecan-2-one		1.22±0.86 a	-	-
Total content of terpenes					9.62±2.57 a	11.38±5.37 b	17.89±3.49 c
94	102488-09-5	25.714	3-Hydroxy-β-damascone		-	1.31±0.07 a	-
95	104-67-6	22.200	2(3H)-Furanone, 5-heptyldihydro-	0.1	-	-	1.52±0.09 a
96	108-94-1	6.314	Cyclohexanone	1.5	5.15±2.12 a	5.07±2.3 a	5.08±1.37 a
97	112-12-9	17.709	2-Undecanone		-	3.68±1.07 a	-
98	1128-08-1	20.013	Dihydrojasmone		-	-	6.00±2.17 a
99	1192-62-7	6.710	Ethanone, 1-(2-furanyl)-		-	1.20±0.13 a	2.50±0.56 b
100	119875-15-9	32.643	3H-Cyclodeca[b]furan-2-one, 4,9-dihydroxy-6-methyl-3,10-dimethylene-3a,4,7,8,9,10,11,11a-octahydro-		1.61±0.75 a	-	-
101	17678-19-2	11.602	Furyl hydroxymethyl ketone		-	-	5.82±1.26 a
102	204648-65-7	23.742	6-Methoxycoumaran-7-ol-3-one		-	11.29±2.34 a	-

103	23726-91-2	20.818	Damascone, β-		-	-	87.62±58.41 a
104	23726-93-4	19.497	2-Buten-1-one, 1-(2,6,6-trimethyl-1,3-cyclohexadien-1-yl)-, (E)-	0.002	44.47±3.54 b	74.74±12.85 c	6.01±0.31 a
105	2460-77-7	22.354	2,5-di-tert-Butyl-1,4-benzoquinone		-	-	5.76±2.37 a
106	35044-68-9	20.134	2-Buten-1-one, 1-(2,6,6-trimethyl-1-cyclohexen-1-yl)-		-	-	4.16±2.34 a
107	3796-70-1	21.781	5,9-Undecadien-2-one, 6,10-dimethyl-, (E)-		6.47±2.71 a	-	-
108	43219-68-7	13.688	Ethanone, 1-(1,4-dimethyl-3-cyclohexen-1-yl)-		-	3.33±2.37 a	-
109	496-16-2	15.580	Benzofuran, 2,3-dihydro-		-	18.04±5.31 a	-
110	502-69-2	30.761	2-Pentadecanone, 6,10,14-trimethyl-		-	2.36±0.75 a	12.06±2.57 b
111	52210-15-8	26.398	2-Cyclohexen-1-one, 4-(3-hydroxy-1-butenyl)-3,5,5-trimethyl-, [R-[R*,R*-(E)]]-		-	1.02±0.08 a	-
112	54344-66-0	24.262	2,5,5,8a-Tetramethyl-6,7,8,8a-tetrahydro-5H-chromen-3-one		-	1.10±0.06 a	-
113	6280-34-8	21.589	5-Cyclohexyl-1H-1,2,3,4-tetrazole		-	6.26±2.73 a	-
Total content of					57.70±16.73 a	129.40±61.32 b	136.53±41.38 c

ketones							
114	87-85-4	26.442	Benzene, hexamethyl-	0.01	14.06±3.71 a	-	-
115	111-65-9	4.178	Octane	2	3.52±1.06 b	-	0.91±0.05 a
116	111-85-3	11.056	Octane, 1-chloro-		1.05±0.08 a	-	-
117	14905-56-7	29.937	Tetradecane, 2,6,10-trimethyl-		10.85±2.37 b	2.40±0.71 a	10.69±2.57 b
118	527-84-4	9.973	o-Cymene		-	1.27±0.61 a	-
119	544-76-3	25.516	Hexadecane		-	5.49±1.27 a	8.12±2.73 b
120	593-49-7	35.819	Heptacosane	0.01	6.19±1.37 b	1.76±0.07 a	7.64±2.71 b
121	629-59-4	20.614	Tetradecane		1.08±0.09 a	1.33±0.04 a	2.35±1.08 b
122	629-78-7	27.777	Heptadecane		-	-	7.75±2.71 a
123	629-92-5	23.132	Nonadecane		5.75±2.41 c	3.44±1.08 b	2.32±0.82 a
124	629-94-7	29.926	Heneicosane		4.55±1.34 a	-	12.52±5.37 b
125	630-04-6	39.330	Hentriacontane		-	2.58±0.27 a	-
Total content of alkanes					47.05±25.31 b	18.27±8.37 a	52.30±34.12 c
126	101-97-3	16.297	Benzeneacetic acid, ethyl ester	400	-	11.15±1.37 a	-
127	106-32-1	15.003	Octanoic acid, ethyl ester	720	10.32±5.37 a	33.26±9.61 b	-
128	106-33-2	25.329	Dodecanoic acid, ethyl ester	0.01	3.97±1.37 a	-	5.980±1.37 b
129	107141-15-1	23.893	Tridecanoic acid, 3-hydroxy-, ethyl ester		3.57±0.86 b	2.25±0.91 a	-
130	110-38-3	29.755	Decanoic acid, ethyl ester	0.002	11.73±3.17 a	15.68±5.37 b	-
131	111-61-5	37.465	Octadecanoic acid, ethyl ester	0.01	1.320±0.08 a	1.310±0.38 a	1.760±0.54 b
132	111-62-6	36.989	Ethyl Oleate	0.1	34.620±6.37 a	168.99±53.73 a	88.220±16.37 b

133	119-36-8	14.909	Methyl salicylate	0.1	39.87±20.31 c	5.79±1.37 a	9.79±2.37 b
134	123-29-5	17.783	Nonanoic acid, ethyl ester		55.64±9.36 c	35.44±5.37 b	21.50±4.37 a
135	123-66-0	9.191	Hexanoic acid, ethyl ester		-	0.85±0.07 a	-
136	124-06-1	29.755	Tetradecanoic acid, ethyl ester		-	14.23±2.37 b	7.710±1.37 a
137	13058-12-3	20.389	2,6-Octadienoic acid, 3,7-dimethyl-, ethyl ester		-	-	17.420±6.31 a
138	131-11-3	21.754	Dimethyl phthalate		-	-	22.920±2.37 a
139	140-11-4	14.000	Acetic acid, phenylmethyl ester	0.05	-	-	5.65±1.37 a
140	14062-18-1	22.964	4-Methoxyphenylacetic acid ethyl ester		-	-	6.47±2.04 a
141	28303-42-6	22.468	Formic acid, dodecyl ester		2.93±1.06 a	-	-
142	544-35-4	36.852	Linoleic acid ethyl ester		21.72±9.37 c	16.140±4.37 b	6.360±1.37 a
143	54546-22-4	33.350	Ethyl 9-hexadecenoate		-	1.89±0.09 a	-
144	5466-77-3	39.565	2-Propenoic acid, 3-(4-methoxyphenyl)-, 2-ethylhexyl ester		-	0.66±0.09 a	-
145	56051-53-7	32.445	Cyclopropanebutanoic acid, 2-[[2-[[2-[(2-pentylcyclopropyl)methyl]cyclopropyl]methyl]cyclopropyl]methyl]-, methyl ester		0.85±0.06 a	0.54±0.16 a	2.05±0.37 b
146	606-45-1	18.799	Benzoic acid, 2-methoxy-, methyl ester		-	-	1.850±0.06 a
147	628-97-7	33.783	Hexadecanoic acid, ethyl		32.04±9.61 b	22.74±8.67 a	54.32±10.37 c

			ester			
148	80060-76-0	36.563	(Z)-18-Octadec-9-enolide	-	6.370±1.37 a	6.99±1.37 a
149	93-89-0	14.245	Benzoic acid, ethyl ester	-	-	46.08±10.37 a
Total content of esters				218.58±37.54 a	337.29±54.38 c	305.07±60.47 b
150	10152-71-3	36.573	Cyclopropaneoctanoic acid, 2-[[2-[(2-ethylcyclopropyl)methyl]cyclopropyl]methyl]-, methyl ester	3.35±0.53 a	-	-
151	104-93-8	9.825	Benzene, 1-methoxy-4-methyl-	-	-	255.35±61.37 a
152	1195-32-0	11.904	Benzene, 1-methyl-4-(1-methylethenyl)-	-	12.07±1.37 a	26.08±2.38 b
153	13217-66-8	5.019	2,2'-Azobis-2-methylpropanimidamide	-	10.57±2.37 a	-
154	13679-41-9	15.768	Furan, 3-phenyl-	8.84±2.06 a	-	-
155	16409-43-1	13.004	2H-Pyran, tetrahydro-4-methyl-2-(2-methyl-1-propenyl)-	-	-	1.16±0.34 a
156	1786-08-9	13.732	2H-Pyran, 3,6-dihydro-4-methyl-2-(2-methyl-1-propenyl)-	-	-	5.39±1.32 a
157	23095-44-5	25.268	Girinimbine	0.28±0.08 a	-	-
158	30364-38-6	19.406	Naphthalene, 1,2-dihydro-1,1,6-trimethyl-	1.85±0.09 a	-	-

159	30364-38-6	19.403	Naphthalene, 1,2-dihydro-1,1,6-trimethyl-	-	5.92±1.06 a	-
160	41678-29-9	16.767	2H-1-Benzopyran, 3,5,6,8a-tetrahydro-2,5,5,8a-tetramethyl-, trans-	-	0.61±0.06 a	-
Total content of other substances				14.32±2.37 a	29.17±6.54 b	287.98±42.91 c
Total content of volatile substances				696.19±63.12 a	763.00±66.73 b	2479.03±203.78 c
